# DEVELOPMENT AND VALIDATION OF THE CLINICAL PHYSICAL RESILIENCE ASSESSMENT SCALE (CHEES) IN OLDER ADULTS

**DOI:** 10.1093/geroni/igae098.2603

**Published:** 2024-12-31

**Authors:** Lina Ma

**Affiliations:** Xuanwu Hospital, Capital Medical University, Beijing, Beijing, China (People’s Republic)

## Abstract

**Objective:**

Physical resilience is an emerging concept that describes an individual’s capacity to recover from stressors. However, few instruments are currently available for assessing physical resilience. We aimed to develop and validate an easy-to-use physical resilience scale, Clinical pHysical rEsilience assEssment Scale (CHEES), in Chinese older adults.

**Methods:**

Delphi method was used to establish an initial scale. Psychometric evaluation of the scale was then tested on 172 older adults. The reliability and validity of CHEES was tested in 1982 community-dwelling and 362 hospitalized older adults. Cronbach’s α coefficient, Spearman-Brown coefficient and Guttman coefficient were used for reliability test. Validity was assessed by content validity and construct validity based on exploratory factor analysis.

**Results:**

After two rounds of expert consultations, three primary and 16 secondary items were determined. Two items were removed after the psychometric evaluation. The final CHEES containing three primary (“intrinsic capacity”, “adapt to change”, and “external support”) and 14 secondary items showed promising properties both in community-dwelling and hospitalized older adults. The acceptance rate of the CHEES was 90.88%, and the qualified rate of the scale was 100%. The Cronbach’s α coefficient of the total scale was 0.826, Spearman-Brown coefficient was 0.737, and Guttman coefficient was 0.731. Exploratory factor analysis extracted three common factors. The factor load of each item was 0.446-0.656, and the cumulative variance contribution rate was 57.49%. Conclusions: CHEES has good reliability and validity, and is suitable for the evaluation of physical resilience of the older adults in both hospital and community.

